# Supplementary material for: Facilitating Access to Current, Evidence-Based Health Information for Non-English Speakers
Source: Healthcare (Basel). 2023 Jul 4;11(13):1932. doi: 10.3390/healthcare11131932 (PMC10340483; doi:10.3390/healthcare11131932)
Supplement: Supplementary file 1 [file healthcare-11-01932-s001.zip › Supplementary Material S1-healthcare-Translated version_PT_PCRS.pdf]

healthcare

MDPI

Supplementary material- Translated version - Portuguese

Perspectiva

Facilitar o acesso a informações atuais em saúde baseadas em evidências para quem não fala inglês

Paulo Henrique Silva Pelicioni [1,2]*, Antonio Michell [3] , Paulo Cezar Rocha dos Santos [4,] and Jennifer Sarah Schulz [5,6,7]

1 School of Health Sciences, University of New South Wales, 2031, Randwick, Australia
2 Neuroscience Research Australia, University of New South Wales, 2031, Randwick, Australia
3 The George Institute for Global Health, Faculty of Medicine and Health, University of New South Wales, 2042, Newtown, Australia; amichell@georgeinstitute.org.au
4 Department of Computer Science and Applied Mathematics, Weizmann Institute of Science, 7632706, Rehovot, Israel; paulocezarr@hotmail.com
5 The Faculty of Law and Justice, University of New South Wales, 2031, Randwick, Australia; jennifer.schulz@unsw.edu.au
6 School of Population Health, University of New South Wales, 2031, Randwick, Australia
7 Faculty of Health and Environmental Sciences, Auckland University of Technology, 0627, Auckland, New Zealand
* E-mail para correspondência: paulo.silvapelicioni@unsw.edu.au.

Resumo: A comunicação científica é fundamental para o desenvolvimento das sociedades e o avanço do conhecimento. No entanto, muitos países e, consequentemente, seus pesquisadores, clínicos e membros da comunidade não têm acesso a essas informações porque elas são divulgadas em inglês e não em seu idioma nativo. Este artigo de perspectiva tem o objetivo de discutir os impactos do problema e também delinear recomendações para facilitar o acesso de pessoas que não falam inglês a informações atuais em saúde baseadas em evidências, ampliando assim o impacto da ciência para além da academia. Primeiro, os autores discutem as barreiras de acesso a informações científicas em relação a saúde para quem não fala inglês e destacam o impacto negativo da imposição do inglês como idioma predominante no meio acadêmico. Em seguida, os autores discutem os impactos da redução do acesso a informações clínicas para quem não fala inglês e como esse acesso reduzido afeta os médicos, os clientes/ pacientes e os sistemas de saúde. Por fim, os autores fornecem recomendações para melhorar o acesso à comunicação científica em todo o mundo.

Palavras-chave: equidade; comunicação em outro idioma; informações sobre saúde

1. Introdução

Embora o inglês não seja o idioma predominante falado em todo o mundo e apenas cerca de 5% da população mundial seja falante nativo de inglês,[1] o inglês é o idioma dominante (ou língua franca) do mundo acadêmico. Nas ciências da saúde e na medicina, a maioria dos periódicos exige o envio de manuscritos em inglês, destacando a predominância do inglês como o principal idioma acadêmico para a comunicação científica.[2] Embora "o inglês possa apresentar a vantagem de permitir que os acadêmicos se comuniquem entre si além das fronteiras e promovam a disseminação global do conhecimento",[3] reconhecemos as desigualdades na comunicação científica, que muitas vezes exclui ou limita o acesso a pessoas que não falam inglês. Esses problemas enfrentados por pessoas que não falam inglês são um anátema para os valores sobre o acesso à saúde e o objetivo de descolonizar a academia,[4] como "não deixar ninguém para

trás" (traduzido do termo "*leave no one behind*").[5-7] "*Leave no one behind*" é um apelo à ação, originalmente contido nos Objetivos de Desenvolvimento Sustentável das Nações Unidas e dos Estados Membros, para criar um mundo mais equitativo no qual as desigualdades, a pobreza e as doenças sejam minimizadas e, por fim, eliminadas.

Para alcançar a equidade na comunicação científica, os acadêmicos sugeriram várias mudanças benéficas, como resumos em outros idiomas além do inglês, conselhos internacionais de editores e versões em idiomas alternativos para algumas revistas.[8,9] O editorial da Lancet em 2019 também reconheceu a necessidade de material científico publicado em outros idiomas além do inglês.[6] Além disso, foi criado um grupo chamado *Healthcare Information for All* (Informações sobre Cuidados de Saúde para Todos, em Português) (HIFA) para mitigar essa necessidade de ter material científico publicado em outros idiomas além do inglês.[1,8]

No entanto, essas sugestões para abordar as desigualdades na comunicação científica ainda não foram implementadas de forma ampla e eficiente. Por exemplo, com a globalização científica, algumas revistas que não falam inglês não aceitam mais manuscritos em seu idioma nativo e a maior parte da literatura relacionada à saúde ainda é publicada somente em inglês. Embora essa abordagem tenha alguma justificativa (por exemplo, as métricas de artigos e periódicos aumentam quando escritos em inglês), ela deixa uma série de pessoas que não falam inglês para trás e excluídas - pesquisadores, clínicos e clientes/pacientes. Além disso, o acesso às informações científicas molda a compreensão da população em relação às medidas de saúde pública e aos tratamentos disponíveis para diferentes doenças; isso pode levar a resultados negativos/negligenciados na saúde de pessoas que não falam ou não têm o inglês como primeiro idioma. Há uma necessidade urgente de resolver esse problema. Portanto, este manuscrito de ponto de vista tem como objetivo discutir o impacto do problema e também delinear recomendações para facilitar o acesso de pessoas que não falam inglês a informações de saúde atuais e baseadas em evidências, ampliando o impacto da ciência para além da academia.

2. Barreiras ao acesso de informações científicas sobre saúde para quem não fala inglês

Nem todos os países falam inglês como idioma nativo. Por exemplo, alguns países da Europa ensinam aos jovens estudantes um segundo idioma. Em algumas universidades europeias, os programas de pós-graduação são em inglês, o que facilita para os alunos adotarem o inglês como o idioma para comunicação no meio acadêmico [10]. Entretanto, nem todos os países têm essa vantagem. Países de renda baixa e média, como Moçambique e Brasil,[7] têm estruturas educacionais diferentes, desde a educação infantil até os programas de pós-graduação. Esses programas não incluem o estudo de habilidades de inglês de alto nível. As taxas caras das escolas de inglês e os problemas de acesso nas regiões rurais criam barreiras para que a população tenha acesso a cursos de inglês nesses países.[11-13] Como as pessoas de países de baixa e média renda que não falam inglês não têm habilidades de alto nível no idioma inglês, elas geralmente buscam acesso a informações usando ferramentas de tradução, como o Google tradutor (*Google Translate*). Infelizmente, a imprecisão do *Google Translate* torna particularmente desafiador a qualidade do acesso ao recebimento de informações relacionadas à saúde.[14]

3. O impacto negativo da imposição do inglês como o idioma "universal" no meio acadêmico

A alta carga de trabalho foi reconhecida como um problema significativo para todos os acadêmicos que falam ou não inglês.[15] No entanto, esses problemas de carga de trabalho foram relatados como sendo piores para os acadêmicos que não falam inglês. Devido às barreiras linguísticas e de acesso, os acadêmicos que não falam inglês levam mais tempo para realizar seu trabalho. Por exemplo, os acadêmicos que não falam inglês

levam mais tempo para redigir suas propostas de financiamento, manuscritos e relatórios em um idioma em que eles não dominam.[13,16] Além disso, devido à sua falta de fluência em inglês, os pesquisadores que não falam inglês às vezes pagam para traduzir seus manuscritos científicos, o que cria dois problemas: (i) esse dinheiro poderia ter sido usado para comprar equipamentos, pagar um funcionário ou oferecer bolsas de estudo;[13] (ii) as informações traduzidas são oferecidas e divulgadas em países onde o inglês é o primeiro idioma, aumentando o número e a diversidade de recursos nesses países. Isso também impõe dois problemas éticos significativos. Primeiro, há um investimento maciço de países que não falam inglês em suas pesquisas, principalmente divulgadas em outro idioma. Em segundo lugar, a população de países que não falam inglês não tem acesso a essas informações devido à barreira do idioma e, portanto, não se beneficia desse conhecimento. Mediante o pagamento de uma taxa, algumas revistas oferecem serviços de tradução por meio de seus editores (Tabela 1).

Table 1. Os 10 principais periódicos da seção "health professions" (profissões da saúde) do Scimago *Journal Rank* que oferecem serviços de tradução, mediante pagamento.

Journal	SJR
The Lancet Digital Health	6,433
British Journal of Sports Medicine	4,764
Qualitative Research in Sport, Exercise and Health	4,045
npj Digital Medicine	3,552
Sports Medicine	3,292
Medical Image Analysis	3,195
International Journal of Behavioral Nutrition and Physical Activity	2,709
Ultrasound in Obstetrics and Gynecology	2,572
Diabetes Technology and Therapeutics	2,374
Journals of Cardiovascular Magnetic Resonance	2,233

Legenda: SJR: é um valor do ranqueamento das revista científicas (Scimago Journal Rank). O valor é proporcional ao peso das citações por documento em cada revista científica. A pontuação media para todos as revistas é de 1,00.

Devido à globalização das informações científicas, as revistas de países que não falam inglês estão mudando seus processos editoriais.[17] Por exemplo, a maioria das revistas indexadas no Brasil não aceita mais manuscritos em português. Essa mudança de política pode refletir as exigências de indexação dos bancos de dados das editoras, segundo as quais os manuscritos devem ser publicados em inglês. Algumas revistas ainda aceitam publicações em inglês e em outros idiomas nativos (Tabela 2); entretanto, as informações não são publicadas em ambos os idiomas, limitando o acesso às informações. Conforme mencionado acima, isso pode ampliar as desigualdades. Um aspecto correlato é que os periódicos aos quais os acadêmicos que não falam inglês poderiam ter acesso estão se tornando extintos, perpetuando a desigualdade na comunicação científica. Essa abordagem restringe o engajamento internacional e se torna uma barreira para a autoria de pesquisadores que têm as habilidades, mas não falam inglês como idioma nativo.[18]

Além disso, as métricas de periódicos publicados em idiomas que não são o inglês costumam ser desfavoráveis devido ao baixo número de citações e alcance.[19,20] O resultado desfavorável, mas provável, é que mais pesquisadores escolherão periódicos escritos apenas em inglês, o que, mais uma vez, agrava o problema.

Table 2. Periódicos da seção profissões da saúde do Scimago *Journal Rank*, parte da *Scientific Electronic Library Online* (SciELO), que publica artigos em inglês e outros idiomas.

Journal	SJR	Language
Acta Ortopédica Brasileira	0,286	Português
CoDAS	0,261	Português/ Espanhol
Revista Brasileira de Ciências do Esporte*	0,216	Português/ Espanhol
Hacia la Promocion de la Salud	0,178	Espanhol
Revista Brasileira de Medicina do Esporte	0,177	Português
Revista Cubana de informacion en Ciencias de la Salud	0,170	Português/ Espanhol
MHSalud	0,150	Espanhol
Jornal Brasileiro de Patologia e Medicina Laboratorial	0,140	Português
Revista Andaluza de Medicina del Deporte	0,140	Português/ Espanhol
Revista Cubana de Farmacia	0,116	Português/ Espanhol
Revista Facultad Nacional de Salud Publica	0,116	Espanhol

Legenda: SJR: é um valor do ranqueamento das revista científicas (Scimago Journal Rank). O valor é proporcional ao peso das citações por documento em cada revista científica. A pontuação media para todos as revistas é de 1,00. *A revista publica somente em português/espanhol em determinados campos de escopo dentro da revista.

4. Acesso reduzido a informações clínicas - impacto sobre os médicos, clientes/pacientes e sistemas de saúde

O idioma e a cultura estão intrinsecamente ligados na maneira como expressamos nossas perspectivas, formulamos nossas perguntas de pesquisa e nos relacionamos com alunos, colegas, pacientes e o público. Toda vez que as informações científicas são divulgadas apenas em inglês, aumentam a desigualdade na área da saúde. Por exemplo, a maioria dos artigos sobre a COVID-19 foi publicada em inglês durante a pandemia.[21] Ao mesmo tempo, a disseminação de notícias falsas e informações falsas foi alarmante em países de baixa e média renda, inclusive em países que não falam inglês.[22-24] Além disso, para quem não fala inglês e depende muito de ferramentas de tradução (que não são totalmente precisas), é um desafio avaliar a confiabilidade e a credibilidade das informações. Além disso, devido à relativa imprecisão das ferramentas de tradução, a interpretação, a disseminação e a aplicação de informações científicas em inglês podem ser tendenciosas para quem não fala inglês. Por fim, a conceituação de termos em inglês no idioma local geralmente é limitada devido a uma gama restrita de expressões que impedem que médicos, pacientes e pesquisadores forneçam informações precisas.

Essas barreiras ao acesso ocorrem em todos os campos relacionados à saúde. Com o atual número reduzido de periódicos que publicam em idiomas diferentes do inglês, os médicos dependem de informações confiáveis, mas que são caras e não estão atualizadas. Pesquisadores e clínicos de todo o mundo escrevem livros sobre diversos tópicos científicos e relacionados à saúde, e esses livros geralmente são traduzidos para idiomas diferentes do inglês quando não são escritos por pessoas que não falam inglês. No entanto, esses livros geralmente abrangem uma gama limitada de questões, às vezes deixando de fora resultados negativos ou nulos, geralmente relatados em revisões sistemáticas e metanálises, publicadas principalmente em inglês. Além disso, quando os autores terminam esses livros, eles não têm uma atualização on-line, o que significa que os leitores

não têm acesso às informações mais recentes.[25] A necessidade de informações atualizadas significa que é improvável que esses clínicos pratiquem cuidados de saúde baseados em evidências recentes e atualizadas. No caso de livros traduzidos, essa necessidade de informações atualizadas é ainda mais crítica. O processo de tradução é normalmente restrito a um número limitado de livros clássicos e geralmente leva vários meses para ser publicado. Assim, as informações contidas nesses livros ficam ainda mais desatualizadas. Isso, por sua vez, pode representar riscos para os resultados de saúde dos indivíduos porque os médicos não têm acesso às evidências científicas atuais. A consequência negativa é que a qualidade e a segurança da assistência médica podem ser prejudicadas.

5. Recomendações

Estamos propondo uma lista não exaustiva de recomendações para começar a abordar os problemas apontados neste artigo:

- Força-tarefa para quem não fala inglês: reconhecemos a importância do HIFA. Mas é necessário criar uma força-tarefa para pessoas que não falam inglês para discutir como divulgar informações científicas relacionadas à saúde para pesquisadores, clínicos e clientes/pacientes e encontrar outras soluções.
- Compreender as necessidades de quem não fala inglês: os pesquisadores devem realizar pesquisas usando questionários e/ou entrevistas para investigar como o acesso às informações para quem não fala inglês pode ser mais equitativo, não apenas para os pesquisadores, mas também para os clínicos e membros da comunidade (como clientes e pacientes). Por exemplo, essa abordagem poderia fornecer aos cientistas dados para iniciar mudanças nos processos editoriais a fim de atender àqueles que precisam de acesso a informações científicas relacionadas à saúde, mas que são impedidos devido a barreiras linguísticas.
- Mudança no tratamento editorial: algumas revistas agora aceitam resumos em outros idiomas além do inglês. Entretanto, os leitores não têm acesso ao artigo completo. Os editores e as revistas poderiam permitir e incentivar os autores que falam outro idioma a enviar seus manuscritos em seu idioma nativo como material suplementar. Além disso, devido ao trabalho adicional exigido dos acadêmicos que não falam inglês para preparar os manuscritos adicionais no seu idioma nativo, as revistas poderiam "compensar" esses autores pelo tempo de tradução do manuscrito para o inglês com um identificador de objeto digital (DOI) diferente. Essa abordagem seria mais justa, reconhecendo o tempo e o esforço para publicar em outros idiomas.
- "Adesão editorial": Editoras como a MDPI poderiam estar abertas a discutir os assuntos mencionados acima. As equipes editoriais de periódicos, como a MDPI, poderiam adotar uma abordagem semelhante. Quando editoras, editores e periódicos renomados adotam essas abordagens, isso pode incentivar outros a adotar processos semelhantes e, assim, reduzir o acesso desigual às informações científicas.

6. Conclusão

Este artigo de perspectiva destacou os desafios criados pelo fato de o inglês ser o idioma predominante no meio acadêmico. Como resultado, há uma série de impactos negativos para pesquisadores e médicos. Em última análise, a qualidade e a segurança da assistência médica podem ser afetadas negativamente porque os médicos / clínicos e pacientes que não falam inglês precisam ter acesso às informações mais recentes baseadas em evidências. Esses problemas prejudicam nossos valores na área da saúde. Por exemplo, a Organização Mundial da Saúde identifica a obtenção da cobertura universal como uma prioridade estratégica com foco na qualidade e na equidade. No entanto, este artigo destacou como as barreiras que populações que não falam inglês enfrentam no acesso a informações científicas impedem o cumprimento dos valores na área da saúde.

Clínicos, pesquisadores e profissionais envolvidos em políticas de saúde enfrentam vários desafios em relação ao conhecimento translacional para comunidades rurais, indígenas e/ou em situação de risco. Para os países que não falam inglês, esses desafios são ampliados pela necessidade de mais acesso e representação no meio acadêmico. Neste manuscrito, enfatizamos a publicação científica; no entanto, o inglês como língua franca também tem um grande impacto na elaboração de subsídios, apresentações em conferências e internacionalização do corpo docente.

Esperamos que as recomendações deste ponto de vista sejam consideradas atentamente para que possamos cumprir nossa aspiração de "não deixar ninguém para trás" ("*leave no one behind*") e, ao mesmo tempo, construir sociedades mais saudáveis em todo o mundo.

Conflitos de interesse: Os autores declaram não haver conflitos de interesse

Contribuições dos autores: Conceitualização, P.H.S.P. e A.M.; redação - preparação do rascunho original, P.H.S.P. e J.S.S.; redação - revisão e edição, P.H.S.P., A.M., P.C.R.S. e J.S.S.; visualização, P.H.S.P., A.M., P.C.R.S. e J.S.S. Tradução para português: P.C.R.S. Tradução para espanhol: A.M Todos os autores leram e concordaram com a versão publicada do manuscrito.

Financiamentos: Paulo Cezar Rocha dos Santos recebe suporte do *Weizmann-IDOR Pioneer Science Fellowship Program*. Os financiadores não interferiram na redação deste manuscrito.

Declaração do Conselho de Ética e Revisão Institucional: Não aplicado.

Termo de consentimento livre e esclarecido: Não aplicado.

Referências

1. 1. Pakenham-Walsh, N. Improving the availability of health research in languages other than English. *Lancet Glob. Health* **2018**, *6*, e1282.
2. Gordin, M.D. *Scientific Babel: How Science Was Done before and after Global English*; University of Chicago Press: Chicago, IL, USA, **2015**.
3. Flowerdew, J. Some thoughts on English for Research Publication Purposes (ERPP) and related issues. *Lang. Teach.* **2015**, *48*, 250–262.
4. Hommes, F.; Monzó, H.B.; Ferrand, R.A.; Harris, M.; Hirsch, L.A.; Besson, E.K.; Manton, J.; Togun, T.; Roy, R.B. The words we choose matter: Recognizing the importance of language in decolonizing global health. *Lancet Glob. Health* **2021**, *9*, e897–e898.
5. Saha, S.; Afrad, M.H.; Saha, S.; Saha, S.K. Towards making global health research truly global. *Lancet Glob. Health* **2019**, *7*, e1175.
6. The Lancet Global Health. The true meaning of leaving no one behind. *Lancet Glob. Health* **2019**, *7*, e553.
7. Baltazar, C.S.; Wheatley, C.; Nsubuga, P. The challenges of getting the research published when English is not the first language: The example of Mozambique Field Epidemiology Training Program. *Pan Afr. Med. J.* **2019**, *33*, 208.
8. Fung, I.C.H. Open access for the non-English-speaking world: Overcoming the language barrier. *Emerg. Themes Epidemiol.* **2008**, *5*, 1.
9. Meneghini, R.; Packer, A.L. Is there science beyond English? Initiatives to increase the quality and visibility of non-English publications might help to break down language barriers in scientific communication. *EMBO Rep.* **2007**, *8*, 112–116.
10. Nashaat-Sobhy, N.; Sanchez-Garcia, D. Lecturers' appraisals of English as a lingua franca in European higher education settings. *J. Psych. Lang. Learn.* **2020**, *2*, 55–72.
11. Li, Y.; Teng, W.; Tsai, L.; Lin, T.M.Y. Does English proficiency support the economic development of non-English-speaking countries? The case of Asia. *Int. J. Educ. Dev.* **2022**, *92*, 102623.
12. Tariq, A.R.; Bilal, H.A.; Sandhu, M.A.; Iqbal, A.; Hayat, U. Difficulties in learning English as a second language in rural areas of Pakistan. *Acad. Res. Int.* **2013**, *4*, 103–113.
13. Ramirez-Castaneda, V. Disadvantages in preparing and publishing scientific papers caused by the dominance of the English language in science: The case of Colombian researchers in biological sciences. *PLoS ONE* **2020**, *15*, e0238372.
14. Patil, S.; Davies, P. Use of Google Translate in medical communication: Evaluation of accuracy. *BMJ* **2014**, *349*, g7392.
15. Pace, F.; D'Urso, G.; Zapulla, C.; Pace, U. The relationship between workload and personal well-being among university professors. *Curr. Psychol.* **2021**, *40*, 3417–3424.
16. Ma, L.P.F. Writing in English as an additional language: Challenges encountered by doctoral students. *High Educ. Res. Dev.* **2021**, *40*, 1176–1190.

17. Baussano, I.; Brzoska, P.; Fedeli, U.; Larouche, C.; Razum, O.; Fung, I.C.H. Does language matter? A case study of epidemiological and public health journals, databases and professional education in French, German and Italian. *Emerg. Themes Epidemiol.* **2008**, *5*, 16.
18. Affun-Adegbulu, C.; Adegbulu, O. Decolonising Global (Public) Health: From Western universalism to Global pluriversalities. *BMJ Glob. Health* **2020**, *5*, e002947.
19. Vinther, S.; Rosenberg, J. Impact factor trends for general medical journals: Non-English-language journals are lagging behind. *Swiss Med. Wkly.* **2012**, *142*, w13572.
20. Di Bitetti, M.S.; Ferreras, J.A. Publish (in English) or perish: The effect on citation rate of using languages other than English in scientific publications. *Ambio* **2017**, *46*, 121–127.
21. Sepulveda-Vidosola, A.C.; Mejla-Arangure, J.M.; Berrera-Cruz, C.; Fuentes-Morales, N.A.; Rodriguez-Zeron, C. Scientific publications during the COVID-19 pandemic. *Arch. Med. Res.* **2020**, *51*, 349–354.
22. Vijaykumar S, Jin Y, Rogerson D, Lu X, Sharma S, Maughan A; et al. How shades of truth and age affect responses to COVID-19 (Mis)information: Randomized survey experiment among WhatsApp users in UK and Brazil. *Nature* **2021**, *8*, 88.
23. Fujita, D.M.; Nali, L.H.S.; Sartori, G.P.; Galisteo, A.J.; Andrade-Junior, H.F.; Luna, E.J.A. Fake news and COVID-19: A concern due to the low vaccine coverage in Brazil. *Saude Soc.* **2022**, *31*, e210298.
24. Biancovilli, P.; Makszin, L.; Jurberg, C. Misinformation on social networks during the novel coronavirus pandemic: A quali-quantitative case study of Brazil. *BMC Public Health* **2021**, *21*, 1200.
25. Savage, W.E.; Olejniczak, A.J. More journal articles and fewer books: Publication practices in the social sciences in the 2010's. *PLoS ONE* **2022**, *17*, e0263410.

Disclaimer/Publisher's Note: The statements, opinions and data contained in all publications are solely those of the individual author(s) and contributor(s) and not of MDPI and/or the editor(s). MDPI and/or the editor(s) disclaim responsibility for any injury to people or property resulting from any ideas, methods, instructions or products referred to in the content.
