# Supplementary material for: Facilitating Access to Current, Evidence-Based Health Information for Non-English Speakers
Source: Healthcare (Basel). 2023 Jul 4;11(13):1932. doi: 10.3390/healthcare11131932 (PMC10340483; doi:10.3390/healthcare11131932)
Supplement: Supplementary file 1 [file healthcare-11-01932-s001.zip › Supplementary Material S2-healthcare-translated-espanol.pdf]

healthcare

MDPI

Punto de Vista

Facilitando el acceso a información de salud actualizada y basada en evidencia para personas que no hablan inglés

Paulo Henrique Silva Pelicioni [1,2]*, Antonio Michell [3] , Paulo Cezar Rocha dos Santos [4,] and Jennifer Sarah Schulz [5,6,7]

1 School of Health Sciences, University of New South Wales, 2031, Randwick, Australia
2 Neuroscience Research Australia, University of New South Wales, 2031, Randwick, Australia
3 The George Institute for Global Health, Faculty of Medicine and Health, University of New South Wales, 2042, Newtown, Australia; amichell@georgeinstitute.org.au
4 Department of Computer Science and Applied Mathematics, Weizmann Institute of Science, 7632706, Rehovot, Israel; paulocezarr@hotmail.com
5 The Faculty of Law and Justice, University of New South Wales, 2031, Randwick, Australia; jennifer.schulz@unsw.edu.au
6 School of Population Health, University of New South Wales, 2031, Randwick, Australia
7 Faculty of Health and Environmental Sciences, Auckland University of Technology, 0627, Auckland, New Zealand
* Correspondencia: paulo.silvapelicioni@unsw.edu.au.

Resumen: La comunicación científica es crucial para el desarrollo de las sociedades y el avance del conocimiento. Sin embargo, muchos países y, en consecuencia, sus investigadores, médicos y miembros de la comunidad carecen de acceso a esta información debido a que se difunde en inglés en lugar de su lengua materna. Este articulo tiene como objetivo analizar los impactos del problema y también esbozar recomendaciones para facilitar el acceso de las personas que no hablan inglés a información de salud actualizada y basada en evidencia, ampliando así el impacto de la ciencia más allá de la academia. En primer lugar, los autores discuten las barreras para acceder a información científica de salud para personas que no hablan inglés y resaltan el impacto negativo de imponer el inglés como idioma predominante en la academia. A continuación, los autores analizan los impactos de un acceso reducido a la información clínica para personas que no hablan inglés y cómo este acceso reducido afecta a los médicos, los clientes y los sistemas de salud. Por último, los autores ofrecen recomendaciones para mejorar el acceso a la comunicación científica en todo el mundo.

Palabras clave: equidad; comunicación en otro idioma; información en salud

1. Introducción

Aunque el inglés no es el idioma predominante a nivel mundial y solo aproximadamente el 5% de la población mundial son hablantes nativos de inglés [1], el inglés es el idioma dominante en la academia (o lengua franca). En ciencias de la salud y medicina, la mayoría de las revistas requieren que los manuscritos se presenten en inglés, lo que destaca el dominio del inglés como el idioma académico principal para la comunicación científica [2]. Aunque "el inglés puede tener la ventaja de permitir a los académicos comunicarse entre sí a través de las fronteras y promover la diseminación global del conocimiento" [3], reconocemos las inequidades en la comunicación científica, que a menudo excluye o limita el acceso a personas que no hablan inglés. Estos problemas que enfrentan las personas que no hablan inglés son contrarios a los valores sobre el acceso a la atención médica y el objetivo de descolonizar la academia [4], como "no dejar a nadie atrás" [5-7]. "No dejar a nadie atrás" es un llamado a la acción, originalmente contenido en los Objetivos de Desarrollo Sostenible de las Naciones Unidas y los Estados Miembros,

para crear un mundo más equitativo en el que se minimicen y, finalmente, se eliminen las desigualdades, la pobreza y las enfermedades.

Para lograr la equidad en la comunicación científica, los académicos han sugerido varios cambios beneficiosos, como la inclusión de resúmenes en idiomas distintos al inglés, la formación de comités editoriales internacionales y la creación de versiones en otros idiomas para algunas revistas [8,9]. The Lancet en 2019 también reconoció la necesidad de publicar material científico publicado en idiomas distintos al inglés [6]. Además, se creó el grupo *Healthcare Information for All* (HIFA) para abordar la necesidad de tener material científico disponible en idiomas distintos al inglés [1,8].

Sin embargo, estas sugerencias para abordar las inequidades en la comunicación científica aún no se han implementado amplia y eficientemente. Por ejemplo, con la globalización científica, algunas revistas no aceptan manuscritos en el idioma nativo de investigadores que no hablan inglés, y la mayoría de la literatura relacionada con la salud sigue siendo publicada solo en inglés. Si bien esta práctica no está completamente sin justificación (por ejemplo, los indicadores para artículos y revistas aumentan cuando se escriben en inglés), deja atrás y excluye a una amplia gama de personas que no hablan inglés, como investigadores, clínicos y clientes/pacientes. Además, el acceso a la información científica moldea la comprensión de la población sobre medidas de salud pública y tratamientos disponibles para diferentes enfermedades, lo que podría llevar a resultados de salud deficientes en personas que no hablan o no tienen el inglés como su primer idioma. Existe una necesidad urgente de abordar este problema. Por lo tanto, este manuscrito tiene como objetivo discutir el impacto del problema y también esbozar recomendaciones para facilitar el acceso de las personas que no hablan inglés a información de salud actualizada y basada en evidencia, extendiendo así el impacto de la ciencia más allá de la academia.

2. Barreras de acceso a la información científica en salud para personas que no hablan inglés

No todos los países hablan inglés como lengua materna. Por ejemplo, algunos países europeos enseñan a los estudiantes un segundo idioma. En algunas universidades europeas, los programas de posgrado se imparten en inglés, lo que facilita que los estudiantes adopten el inglés como el idioma dominante en el ámbito académico [10]. Sin embargo, no todos los países tienen esta ventaja. Los países de ingresos bajos y medianos, como Mozambique y Brasil [7], tienen estructuras educativas diferentes, desde la educación temprana hasta los programas de posgrado. Estos programas no incluyen el estudio de habilidades avanzadas de inglés. Las altas tarifas de las escuelas de inglés y los problemas de acceso en las zonas rurales crean barreras para las personas que no hablan inglés en estos países [11-13]. Dado que las personas que no hablan inglés en países de ingresos bajos y medianos no tienen un nivel avanzado de inglés, a menudo buscan acceso a información utilizando herramientas de traducción como *Google Translate*. Desafortunadamente, la falta de precisión de *Google Translate* dificulta especialmente recibir información apropiada relacionada con la salud [14].

3. El impacto negativo de imponer el inglés como el idioma "universal" en la academia

La alta carga de trabajo se ha reconocido como un problema significativo tanto para los académicos que hablan inglés como para los que no lo hablan [15]. Sin embargo, los autores argumentan que estos problemas de carga de trabajo son aún peores para los académicos que no hablan inglés. Debido a las barreras del idioma y el acceso, a los académicos que no hablan inglés les lleva más tiempo llevar a cabo su trabajo. Por ejemplo, a los no angloparlantes les lleva más tiempo escribir sus propuestas de financiamiento, manuscritos e informes en un idioma diferente al nativo [13,16]. Además, debido a su falta de fluidez en inglés, los investigadores que no hablan inglés a veces pagan para traducir sus manuscritos científicos, lo que crea dos problemas: (i) este dinero

podría haberse utilizado en su lugar para comprar equipos, pagar a un empleado u ofrecer becas [13]; (ii) la información traducida se ofrece y se difunde en países donde el inglés es el primer idioma, lo que aumenta la cantidad y diversidad de recursos en estos países. Esto plantea también dos problemas éticos significativos. En primer lugar, hay una inversión masiva por parte de los países no anglófonos en su investigación, que se disemina principalmente en otro idioma. En segundo lugar, la población de los países no anglófonos no tiene acceso a esta información debido a la barrera del idioma y, por lo tanto, no se beneficia de este conocimiento. Algunas revistas ofrecen servicios de traducción a través de sus editores por un costo (Tabla 1).

Table 1. Top 10 revistas en la sección de "*health professions*" del Scimago *Journal Rank* que ofrecen servicios de traducción por un costo.

Journal	SJR
The Lancet Digital Health	6,433
British Journal of Sports Medicine	4.764
Qualitative Research in Sport, Exercise and Health	4.045
npj Digital Medicine	3.552
Sports Medicine	3.292
Medical Image Analysis	3.195
International Journal of Behavioral Nutrition and Physical Activity	2.709
Ultrasound in Obstetrics and Gynecology	2.572
Diabetes Technology and Therapeutics	2.374
Journals of Cardiovascular Magnetic Resonance	2.233

Leyenda: SJR es un valor del Scimago *Journal Rank*. La puntuación está relacionada con las citas ponderadas por documento en cada revista. La puntuación SJR promedio para todas las revistas es 1.00.

Debido a la globalización de la información científica, las revistas en países de habla no inglesa están cambiando sus procesos editoriales [17]. Por ejemplo, la mayoría de las revistas indexadas en Brasil ya no aceptan manuscritos en portugués. Este cambio de política puede reflejar las demandas de indexación de las bases de datos de los editores, donde los manuscritos deben ser publicados en inglés. Algunas revistas aún aceptan publicaciones en inglés y otros idiomas nativos (Tabla 2); sin embargo, la información no se publica en ambos idiomas, lo que limita el acceso a la información. Como se mencionó anteriormente, esto puede magnificar las desigualdades. Como resultado, las revistas a las que los académicos no anglófonos podrían acceder están desapareciendo, perpetuando la inequidad en la comunicación científica. Este enfoque restringe el compromiso internacional y se convierte en una barrera para la autoría de los investigadores que tienen las habilidades, pero no hablan inglés como lengua materna [18]. Además, las métricas de las revistas publicadas en idiomas no ingleses a menudo son desfavorables debido a las citas y el alcance limitados [19,20]. La lamentable pero probable consecuencia es que más investigadores elegirán revistas escritas solo en inglés, lo que, nuevamente, exacerba el problema.

Table 2. Revistas en la sección de "*health professions*" del Scimago Journal Rank que forman parte de la Biblioteca Científica Electrónica en Línea (SciELO) y que publican artículos en inglés y otros idiomas.

Journal	SJR	Language
Acta Ortopédica Brasileira	0.286	Portugués
CoDAS	0.261	Portugués/ Español
Revista Brasileiras de Ciências do Esporte	0.216	Portugués/ Español *
Hacia la Promocion de la Salud	0.178	Español
Revista Brasileira de Medicina do Esporte	0.177	Portugués
Revista Cubana de informacion en Ciencias de la Salud	0.170	Portugués/ Español
MHSalud	0.150	Español
Jornal Brasileiro de Patologia e Medicina Laboratorial	0.140	Portugués
Revista Andaluza de Medicina del Deporte	0.140	Portugués/ Español
Revista Cubana de Farmacia	0.116	Portugués/ Español
Revista Facultad Nacional de Salud Publica	0.116	Español

Leyenda: SJR es un valor del Scimago Journal Rank. La puntuación está relacionada con las citas ponderadas por documento en cada revista. La puntuación SJR promedio para todas las revistas es 1.00. *La revista publica únicamente en portugués/español en ciertos campos de Scopus dentro de la revista.

4. La reducción del acceso a la información clínica tiene impactos significativos en los clínicos, los clientes y los sistemas de salud.

El lenguaje y la cultura están intrínsecamente vinculados, ya que influyen en cómo expresamos nuestras perspectivas, enmarcamos nuestras preguntas de investigación y nos relacionamos con estudiantes, colegas, pacientes y el público en general. Cada vez que se disemina información científica solo en inglés, se incrementa la inequidad en la atención médica. Por ejemplo, la mayoría de los artículos sobre COVID-19 se publicaron en inglés durante la pandemia [21]. Al mismo tiempo, la difusión de noticias falsas y desinformación fue alarmante en países de ingresos bajos y medianos, incluyendo aquellos de habla no inglesa [22-24]. Además, para aquellos que no hablan inglés y dependen en gran medida de herramientas de traducción (que no son totalmente precisas), resulta desafiante evaluar la confiabilidad y credibilidad de la información. Asimismo, debido a la relativa inexactitud de las herramientas de traducción, la interpretación, difusión y aplicación de la información científica en inglés puede estar sesgada para aquellos que no hablan inglés. Por último, la conceptualización de términos en inglés en el lenguaje local suele ser limitada debido a una gama restringida de expresiones, lo que dificulta que los profesionales de la salud, los pacientes y los investigadores brinden información precisa.

Estas barreras de acceso se presentan en todos los campos relacionados con la salud. Con el reducido número actual de revistas que publican en idiomas distintos al inglés, los clínicos dependen de información confiable pero costosa y desactualizada. Investigadores y clínicos de todo el mundo escriben libros sobre diversos temas científicos y relacionados con la salud, y estos libros a menudo se traducen a otros idiomas cuando no son escritos por personas de habla inglesa. Sin embargo, estos libros suelen abarcar una gama limitada de temas, a veces omitiendo resultados negativos o nulos, que a menudo se informan en revisiones sistemáticas y metanálisis, publicados principalmente en inglés. Además, cuando los autores finalizan estos libros, no cuentan con una actualización en línea, lo que significa que los lectores no tienen acceso a la información más reciente [25]. La necesidad de contar con información actualizada significa que es poco probable que estos clínicos practiquen una atención médica basada en evidencia. En el caso de los libros traducidos,

esta necesidad de información actualizada es aún más crítica. El proceso de traducción generalmente se limita a un número reducido de libros clásicos y suele llevar varios meses publicarlos. Por lo tanto, la información en esos libros está aún más desactualizada. Esto, a su vez, puede representar riesgos para los resultados de salud de las personas, ya que los clínicos no tienen acceso a la evidencia científica actual. La lamentable consecuencia es que la calidad y seguridad de la atención médica pueden verse comprometidas.

5. Recomendaciones

A continuación se presenta una lista no exhaustiva de recomendaciones para comenzar a abordar los problemas descritos en este artículo:

- Grupo de trabajo para hablantes no nativos de inglés: reconocemos la importancia de HIFA. Sin embargo, existe la necesidad de crear un grupo de trabajo para hablantes no nativos de inglés que discuta cómo difundir información científica relacionada con la salud a investigadores, clínicos y clientes/pacientes, y encontrar otras soluciones.
- Comprender las necesidades de los hablantes no nativos de inglés: los investigadores deben realizar investigaciones utilizando encuestas y/o entrevistas para investigar cómo se podría lograr un acceso más equitativo a la información para los hablantes no nativos de inglés, no solo para los investigadores, sino también para los clínicos y miembros de la comunidad (por ejemplo, clientes y pacientes). Este enfoque podría proporcionar a los científicos datos para iniciar cambios en los procesos editoriales que apunten a aquellos que necesitan acceder a información científica relacionada con la salud pero que se ven obstaculizados debido a las barreras del idioma.
- Cambios en el manejo editorial: algunas revistas ahora aceptan resúmenes en idiomas distintos al inglés. Sin embargo, los lectores no tienen acceso al artículo completo. Los editores y las revistas podrían permitir y alentar a los autores que hablan otro idioma a enviar sus manuscritos en su idioma nativo como material suplementario. Además, debido al trabajo adicional que los académicos no nativos de inglés deben realizar para preparar los manuscritos, las revistas podrían "compensar" a estos autores por el tiempo que les lleva traducir el manuscrito al inglés con un identificador de objeto digital (DOI) diferente. Este enfoque sería más equitativo, reconociendo el tiempo y esfuerzo requeridos para publicar en otros idiomas.
- "Compromiso editorial": Editores como MDPI podrían estar dispuestos a discutir los asuntos mencionados anteriormente. Los equipos editoriales de revistas, como MDPI, podrían adoptar un enfoque similar. Cuando editores, revistas y publicadores reconocidos toman este tipo de medidas, pueden alentar a otros a adoptar procesos similares y, de esta manera, reducir el acceso inequitativo a la información científica.

6. Conclusión

En conclusión, este artículo de opinión ha expuesto los desafíos derivados de la predominancia del inglés como idioma principal en la academia. Los impactos negativos afectan a investigadores, clínicos y a la calidad general de la atención médica. Las barreras que enfrentan los hablantes no nativos de inglés para acceder a la información científica socavan los valores de la atención médica, como lograr cobertura universal, calidad y equidad. Es fundamental abordar estas barreras y trabajar para garantizar un acceso equitativo a información basada en evidencia para todos, independientemente de su dominio del idioma. Mediante la implementación de las estrategias recomendadas y fomentando la colaboración entre los diferentes actores, podemos avanzar hacia una comunicación científica más inclusiva y equitativa.

Los clínicos, investigadores y responsables de políticas de salud enfrentan varios desafíos en cuanto la transferencia del conocimiento para comunidades rurales, indígenas y/o en situación de riesgo. Para los países de habla no inglesa, estos desafíos se amplifican debido a la necesidad de tener un mayor acceso y representación en la academia. En este manuscrito, hemos enfatizado la publicación científica; sin embargo, el inglés como lingua

franca también tiene un impacto importante en la redacción de propuestas de financiamiento, presentaciones en conferencias e internacionalización del cuerpo docente.

Esperamos que las recomendaciones presentadas en este punto de vista sean consideradas cuidadosamente, para así poder cumplir con nuestra aspiración de "no dejar a nadie atrás" mientras construimos sociedades más saludables en todo el mundo.

Conflictos de intereses: Los autores declaran no tener conflictos de intereses.

Contribución de los autores: Conceptualización, P.H.S.P. y A.M.; redacción del borrador original, P.H.S.P. y J.S.S.; revisión y edición del texto, P.H.S.P., A.M., P.C.R.S. y J.S.S.; visualización, P.H.S.P., A.M., P.C.R.S. y J.S.S. Traducción para portugués: P.C.R.S. Traducción para español: A.M Todos los autores han leído y aceptado la versión publicada del manuscrito.

Financiamiento: Paulo Cezar Rocha dos Santos recebe suporte do Weizmann-IDOR Pioneer Science Fellowship Program. Os financiadores no interferirán en la escrita del artículo.

Declaración del Comité de Ética Institucional: No aplica.

Declaración de consentimiento informado: No aplica.

Referencias

1. Pakenham-Walsh, N. Improving the availability of health research in languages other than English. *Lancet Glob. Health* **2018**, *6*, e1282.
2. Gordin, M.D. *Scientific Babel: How Science Was Done before and after Global English*; University of Chicago Press: Chicago, IL, USA, **2015**.
3. Flowerdew, J. Some thoughts on English for Research Publication Purposes (ERPP) and related issues. *Lang. Teach.* **2015**, *48*, 250–262.
4. Hommes, F.; Monzó, H.B.; Ferrand, R.A.; Harris, M.; Hirsch, L.A.; Besson, E.K.; Manton, J.; Togun, T.; Roy, R.B. The words we choose matter: Recognizing the importance of language in decolonizing global health. *Lancet Glob. Health* **2021**, *9*, e897–e898.
5. Saha, S.; Afrad, M.H.; Saha, S.; Saha, S.K. Towards making global health research truly global. *Lancet Glob. Health* **2019**, *7*, e1175.
6. The Lancet Global Health. The true meaning of leaving no one behind. *Lancet Glob. Health* **2019**, *7*, e553.
7. Baltazar, C.S.; Wheatley, C.; Nsubuga, P. The challenges of getting the research published when English is not the first language: The example of Mozambique Field Epidemiology Training Program. *Pan Afr. Med. J.* **2019**, *33*, 208.
8. Fung, I.C.H. Open access for the non-English-speaking world: Overcoming the language barrier. *Emerg. Themes Epidemiol.* **2008**, *5*, 1.
9. Meneghini, R.; Packer, A.L. Is there science beyond English? Initiatives to increase the quality and visibility of non-English publications might help to break down language barriers in scientific communication. *EMBO Rep.* **2007**, *8*, 112–116.
10. Nashaat-Sobhy, N.; Sanchez-Garcia, D. Lecturers' appraisals of English as a lingua franca in European higher education settings. *J. Psych. Lang. Learn.* **2020**, *2*, 55–72.
11. Li, Y.; Teng, W.; Tsai, L.; Lin, T.M.Y. Does English proficiency support the economic development of non-English-speaking countries? The case of Asia. *Int. J. Educ. Dev.* **2022**, *92*, 102623.
12. Tariq, A.R.; Bilal, H.A.; Sandhu, M.A.; Iqbal, A.; Hayat, U. Difficulties in learning English as a second language in rural areas of Pakistan. *Acad. Res. Int.* **2013**, *4*, 103–113.
13. Ramirez-Castaneda, V. Disadvantages in preparing and publishing scientific papers caused by the dominance of the English language in science: The case of Colombian researchers in biological sciences. *PLoS ONE* **2020**, *15*, e0238372.
14. Patil, S.; Davies, P. Use of Google Translate in medical communication: Evaluation of accuracy. *BMJ* **2014**, *349*, g7392.
15. Pace, F.; D'Urso, G.; Zapulla, C.; Pace, U. The relationship between workload and personal well-being among university professors. *Curr. Psychol.* **2021**, *40*, 3417–3424.
16. Ma, L.P.F. Writing in English as an additional language: Challenges encountered by doctoral students. *High Educ. Res. Dev.* **2021**, *40*, 1176–1190.
17. Baussano, I.; Brzoska, P.; Fedeli, U.; Larouche, C.; Razum, O.; Fung, I.C.H. Does language matter? A case study of epidemiological and public health journals, databases and professional education in French, German and Italian. *Emerg. Themes Epidemiol.* **2008**, *5*, 16.
18. Affun-Adegbulu, C.; Adegbulu, O. Decolonising Global (Public) Health: From Western universalism to Global pluriversalities. *BMJ Glob. Health* **2020**, *5*, e002947.
19. Vinther, S.; Rosenberg, J. Impact factor trends for general medical journals: Non-English-language journals are lagging behind. *Swiss Med. Wkly.* **2012**, *142*, w13572.
20. Di Bitetti, M.S.; Ferreras, J.A. Publish (in English) or perish: The effect on citation rate of using languages other than English in scientific publications. *Ambio* **2017**, *46*, 121–127.

21. Sepulveda-Vidosola, A.C.; Mejla-Arangure, J.M.; Berrera-Cruz, C.; Fuentes-Morales, N.A.; Rodriguez-Zeron, C. Scientific publications during the COVID-19 pandemic. *Arch. Med. Res.* **2020**, *51*, 349–354.
22. Vijaykumar S, Jin Y, Rogerson D, Lu X, Sharma S, Maughan A; et al. How shades of truth and age affect responses to COVID-19 (Mis)information: Randomized survey experiment among WhatsApp users in UK and Brazil. *Nature* **2021**, *8*, 88.
23. Fujita, D.M.; Nali, L.H.S.; Sartori, G.P.; Galisteo, A.J.; Andrade-Junior, H.F.; Luna, E.J.A. Fake news and COVID-19: A concern due to the low vaccine coverage in Brazil. *Saude Soc.* **2022**, *31*, e210298.
24. Biancovilli, P.; Makszin, L.; Jurberg, C. Misinformation on social networks during the novel coronavirus pandemic: A quali-quantitative case study of Brazil. *BMC Public Health* **2021**, *21*, 1200.

25. Savage, W.E.; Olejniczak, A.J. More journal articles and fewer books: Publication practices in the social sciences in the 2010's. *PLoS ONE* **2022**, *17*, e0263410.

Descargo de responsabilidad/Nota del editor: Las afirmaciones, opiniones y datos contenidos en todas las publicaciones son únicamente responsabilidad del autor(es) y colaborador(es) individual(es) y no de MDPI y/o del editor(es). MDPI y/o el editor(es) renuncian a toda responsabilidad por cualquier daño a personas o propiedades que resulte de las ideas, métodos, instrucciones o productos mencionados en el contenido.
